# Demonstration of the test-retest reliability and sensitivity of the Lower Limb Functional Index-10 as a measure of functional recovery post burn injury: a cross-sectional repeated measures study design

**DOI:** 10.1186/s41038-016-0043-y

**Published:** 2016-06-06

**Authors:** Margaret E. Ryland, Tiffany L. Grisbrook, Fiona M. Wood, Michael Phillips, Dale W. Edgar

**Affiliations:** 1 School of Physiotherapy and Exercise Science, Curtin University, Bentley, 6102 WA Australia; 2 Fiona Wood Foundation of Western Australia, Fiona Stanley Hospital, 11 Warren Drive, Murdoch, 6150 WA Australia; 3 State Adult Burn Unit, Fiona Stanley Hospital, Level 4, 11 Warren Smith Drive, Murdoch, 6150 WA Australia; 4 Burn Injury Research Unit, The University of Western Australia, Crawley, 6009 WA Australia; 5 Harry Perkins Institute of Medical Research, The University of Western Australia, Crawley, 6009 WA Australia; 6 Burn Injury Research Node, The University of Notre Dame Australia, 19 Mouat St, Fremantle, 6160 WA Australia

**Keywords:** Outcome measure, Questionnaire, Lower limb function, Internal consistency, Minimal detectable change, Burn

## Abstract

**Background:**

Lower limb burns can significantly delay recovery of function. Measuring lower limb functional outcomes is challenging in the unique burn patient population and necessitates the use of reliable and valid tools. The aims of this study were to examine the test-retest reliability, sensitivity, and internal consistency of Sections 1 and 3 of the Lower Limb Functional Index-10 (LLFI-10) questionnaire for measuring functional ability in patients with lower limb burns over time.

**Methods:**

Twenty-nine adult patients who had sustained a lower limb burn injury in the previous 12 months completed the test-retest procedure of the study. In addition, the minimal detectable change (MDC) was calculated for Section 1 and 3 of the LLFI-10. Section 1 is focused on the activity limitations experienced by patients with a lower limb disorder whereas Section 3 involves patients indicating their current percentage of pre-injury duties.

**Results:**

Section 1 of the LLFI-10 demonstrated excellent test-retest reliability (intra-class correlation coefficient (ICC) 0.98, 95 % CI 0.96–0.99) whilst Section 3 demonstrated high test-retest reliability (ICC 0.88, 95 % CI 0.79–0.94). MDC scores for Sections 1 and 3 were 1.27 points and 30.22 %, respectively. Internal consistency was demonstrated with a significant negative association (*r*_*s*_ = −0.83) between Sections 1 and 3 of the LLFI-10 (*p* < 0.001).

**Conclusions:**

This study demonstrates that Section 1 and 3 of the LLFI-10 are reliable for measuring functional ability in patients who have sustained lower limb burns in the previous 12 months, and furthermore, Section 1 is sensitive to changes in patient function over time.

## Background

Over the past decade, there has been increasing attention on the use of outcome measures in burn rehabilitation to evaluate patients’ overall function [1]. Following a burn, a major goal of rehabilitation is to restore the patient’s pre-injury function in all aspects of their daily life [2]. The use of valid, reliable, and sensitive outcome measures which evaluate a patient’s level of function, and thus recovery, is therefore integral to treatment planning, evaluating the effectiveness of interventions, and monitoring patient recovery [3]. Inclusion of outcome measures at all levels of the International Classification of Functioning, Disability and Health (ICF) [1] is preferred, to provide an indication of patient recovery encompassing all aspects of functioning [4]. In the burn population, both generic and burn-specific outcome measures provide information regarding a patient’s level of function. As burn location has been shown to influence burn rehabilitation [5], outcome measures that are specific to the location of a burn may be of additional value. Consequently, choosing a range of outcome measures aids effective assessment across the spectrum of functional recovery in patients with burns.

Assessment and management of activities and participation are integral components of the recovery of function according to the ICF [1]. Individuals with burn injuries experience a number of activity limitations and participation restrictions. Frequently documented activity limitations in burn-injured patients include hand function, mobility, activities of daily living (ADL), and sexuality [6]. Whilst upper limb burn injuries are far more common than lower limb burn injuries [7], lower limb burns have been shown to be associated with significant delay in patient recovery to pre-injury levels of function in comparison to upper limb burns [8]. Given the association between burn location and patient rehabilitation post burn injury, outcome measures utilized to assess activity limitations and participation restrictions should also take burn location into account.

The shortened disability of the arm, shoulder and hand (QuickDASH) questionnaire measures one’s ability to perform ADL, as well as work- and leisure-related activities that require the use of the upper limbs [9]. It has been demonstrated to be valid, reliable, and sensitive in patients with upper limb burn injuries [10]. A similar measure of activity limitation specific to the lower limb is the Lower Limb Functional Index (LLFI) [11]. The LLFI questionnaire was developed by Gabel et al. in 2012 as a patient self-report questionnaire designed to assess the function of patients with various lower limb musculoskeletal disorders [11]. Furthermore, Spanish and Turkish versions of the LLFI have also been published and validated [12, 13]. Patient self-report questionnaires are particularly useful options in Western Australia (WA) and other large states that employ Telehealth services to follow up patients who are not always able to return to the Burn Unit where they were initially managed. The LLFI was initially developed following recognition of the limitations in previously used outcome measures including the Lower Extremity Functional Scale (LEFS) [11]. In comparison to the LEFS, the LLFI was shown to have greater efficiency, a lower error response rate, and also greater sensitivity or responsiveness to change [11]. Since its development, the LLFI has been shown to be valid and reliable in a patient population with various lower limb musculoskeletal disorders including hip osteoarthritis, hamstring muscle strain, and patellar fracture [11]. An abbreviated version, the Lower Limb Functional Index-10 (LLFI-10) [14] has been shown to be a valid measure of lower limb function and activity limitation in patients with lower limb burn injury [14, 15]. The test-retest reliability of the LLFI-10 has not previously been investigated in any patient population however is of interest in the burn patient population due to the complex nature of burn injuries and the possibility for pain levels to fluctuate over time [16].

This study aimed to examine the test-retest reliability, sensitivity, and internal consistency of Section 1 and 3 of the LLFI-10 in patients with lower limb burn injuries in WA. We hypothesized that Section 1 and 3 would demonstrate a high level of test-retest reliability, a level of sensitivity similar to the LEFS in patients with musculoskeletal conditions, and a high level of internal consistency for patients with lower limb burns.

## Methods

### Patients

Adult patients with a lower limb burn injury attending the Western Australian State Adult Burn Unit outpatient clinic between the 17th of March 2015 and the 30th of March 2015 were approached for recruitment to this study. All patients recruited provided written informed consent were aged 18 years or over, and had sustained a lower limb burn injury within the previous 12 months. For the purposes of this study, we defined lower limb burn injury to include burns affecting the thigh, buttocks, lower leg, ankle, and foot. Patients were excluded if they were not competent in written and spoken English, had concomitant psychological issues, or had open or unhealed wounds as confirmed by a senior burn nurse or surgeon.

### Outcome measure

The 10-item short form of the LLFI, the LLFI-10 questionnaire, was developed from the original 25-item LLFI questionnaire [11, 15]. It consists of the stand-alone essential 10 items and three optional sections that evaluate the patient’s function at the time of assessment [15]. Section 1 of the LLFI-10 contains 10 items relating to the activity limitations experienced by patients with a lower limb disorder, such as “I have difficulty bending, squatting and/or reaching down”. The three possible responses to each item are “Yes” scored 1 point, “Sometimes” scored 0.5 point, and “No” scored 0 point. A higher total score on Section 1 represents *poorer* function. Items 1–6 of the LLFI-10 assess a patient’s health-related quality of life whereas items 7–10 of the LLFI-10 assess lower extremity dysfunction. Section 2, the Patient Specific Index, is designed to interpret qualitative information and requires the patient to list five activities that they feel have been affected by their lower limb disorder. Each activity is then scored from 0 (never affected/can do activity normally) to 10 (always affected/cannot do activity at all). Section 3 of the LLFI-10 involves patients indicating their current percentage of pre-injury duties, with scores ranging from 0 to 100 % where 100 % represents full performance of their pre-injury duties. An advantage of Section 3 is that it requires patients to respond to one question only, and thus, completion is not a significant burden on patients. The final section on the LLFI-10 (Section 4) asks patients to rank their overall status on a scale from 0 to 10 where 10 represents the worst possible status [15].

Recently published data from the WA burn population indicated that Section 1 provides the most useful information regarding a patient’s functional abilities and Section 3 offers a brief, useful assessment of current overall functional ability [14]. Section 2, 3, and 4 are suggested to be optional sections of the LLFI-10 where Section 2 provides individualized, qualitative information about a patient’s activity limitations [15]. Section 3 and 4 are similar single-item scales that provide a quantitative measure of a patient’s perceived recovery status. Due to the similarity between Section 3 and 4, there is the potential for redundancy. Information gained from Section 1 and 3 were thus included in data analysis for this study.

### Procedure

Recruitment of a sample of 38 patients was planned in order to adequately power the test-retest analysis procedure (*α* = 0.05, *β* = 0.2), to allow for loss of patient follow-up after assessment time point 1, and to accommodate a recruitment period of 5 weeks [17]. A minimum of 27 patients were required to adequately power a repeated measures analyses with two-paired data points with 95 % confidence limits [17]. Patients received the LLFI-10 questionnaire to complete on attendance at their outpatient clinic appointment. Patients were provided with a second LLFI-10 questionnaire to complete ideally 24 h following the previous questionnaire and return by mail in a stamped addressed envelope provided. Second questionnaires were included in analysis if the written date of completion on the questionnaire was within 48 h of the completion of the previous questionnaire. A maximum period of 48 h was specifically chosen to account for the possibility of patients with recent burns experiencing rapid improvement. No specific treatment was provided to the patients included in this study between the two time points, and thus, no changes in patient function were expected. Patient demographics and individual burn characteristics including age, gender, percentage of total body surface area (%TBSA) burned, burn depth, and burn location were recorded from medical files accessed in the Burn Outpatient Clinic. This study was approved by the Royal Perth Hospital Human Research Ethics Committee (Ethics approval number 14-146) and Curtin University Human Research Ethics Committee (Ethics approval number HR228/2014).

### Data analysis

All data analysis was conducted using Stata Statistical Software, release 13 (StataCorp, LP, 2013, College Station, TX). Descriptive analyses were completed and are presented using medians and ranges, unless otherwise stated. The Shapiro-Wilk test was used to assess data normality. Wilcoxon Signed-rank tests were used to determine if there were any significant differences in patient demographics between the two assessment time points due to patients being lost to follow-up. The alpha level was set to *p* < 0.05.

Test-retest reliability of Sections 1 and 3 was assessed by calculating the intra-class correlation coefficient (ICC) using a two-way random effects model. The level of significance was set at *p* < 0.05, and an ICC value <0.75 was interpreted as moderate, 0.75–0.89 as high, and ≥0.90 as excellent [18].

To assess the sensitivity of the LLFI-10 to detect changes in lower limb function, the minimal detectable change (MDC) was calculated for both Sections 1 and 3 of the questionnaire using initial and follow-up scores. The MDC represents the degree of real change able to be measured by a test with 95 % certainty and is calculated using the following equation: MDC = 1.96 × √2 × SEM [19], where SEM is the standard error of measurement and is calculated using the following equation: SEM = SD √(1−*R*) [19], where *R* = test-retest reliability (ICC).

Internal consistency of Section 1 and 3 of the LLFI-10 was investigated on patients using data from questionnaires with both sections completed at time point 1 and time point 2. Internal consistency assesses the extent to which two different items on a test or questionnaire measure the same variable [20]. Thus, in the current study, internal consistency was assessed by correlating Section 1 total scores and Section 3 percentages of pre-injury duties in order to determine the extent to which these two sections measure a patient’s functional ability. Spearman’s rank coefficient was used to assess the correlation between Section 1 and 3 of the LLFI-10. The Spearman’s rho (*r*_*s*_) was interpreted as very high (1.00–0.90), high (0.90–0.70), moderate (0.70–0.50), low (0.50–0.30), or negligible correlation (<0.30) [21].

## Results

### Patient characteristics

**Fig. 1:**
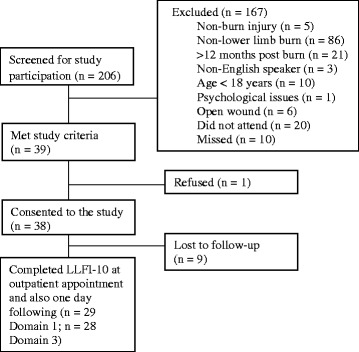
Flow of recruitment of participants of the study

A total of 38 adult patients (21 males, 17 females), aged 18–79 years, who had sustained a lower limb burn injury within the previous year were recruited for this study. Only 29 patients completed Section 1, and 28 patients completed Section 3 of the LLFI-10 at time point 2. Therefore, only patients with a complete dataset were included in the test-retest reliability and sensitivity analysis procedures. The flow of recruitment of participants in this study is detailed in Fig. 1. There were no significant differences in patient demographics between those who completed the questionnaire at both time points and those lost to follow-up in regard to age and %TBSA burned (*p* = 0.474 and *p* = 0.995, respectively).

Demographic and clinical characteristics of all patients, at both time points, are described in Table 1. Thirty-one patients (82 %) of those recruited for the study had sustained a minor burn, defined as %TBSA burned ≤15 % [22]. The majority of patients recruited sustained mid-dermal (37 %) or deep dermal burn injuries (40 %). The mean length of time since burn injury to LLFI assessment was 95 days (4–366 days). In the sample for final analysis, 24 % of patients had sustained a burn injury to their left leg, 38 % to their right leg, and 38 % to both legs.

**Table 1:** Characteristics of patients at time points 1 and 2

Characteristic	Time point 1 (*n* = 38)	Time point 2 (*n* = 29)
Age (years) (median (IQR))	35 (25–49)	36 (27–50.5)
% TBSA burned (median (IQR))	2.8 (1–7.7)	2.2 (0.9–11.3)
Minor burn (*n* (%))	31 (81.6)	22 (75.9)
Male (*n* (%))	21 (55.3)	15 (51.7)
Surgery (*n* (%))	26 (68.4)	19 (65.5)
Superficial dermal burn (*n* (%))	3 (7.9)	2 (6.9)
Mid dermal burn (*n* (%))	14 (36.8)	10 (34.5)
Deep dermal burn (*n* (%))	15 (39.5)	12 (41.4)
Full-thickness burn (*n* (%))	6 (15.8)	5 (17.2)
Left leg affected (*n* (%))	7 (18.4)	7 (24.1)
Right leg affected (*n* (%))	16 (42.1)	11 (37.9)
Both legs affected (*n* (%))	15 (39.5)	11 (37.9)

*IQR* interquartile range, *TBSA* total body surface area, *n* number of participants

### Test-retest reliability and sensitivity

The mean total score on Section 1 and percentage of ability to perform normal duties on Section 3 for the sample population for final analysis at time points 1 and 2 are presented in Table 2. Test-retest reliability analyses on total scores in Section 1 and percentages of normal duties in Section 3 of the LLFI-10 gave an ICC (95 % confidence interval (CI)) of 0.98 (0.96–0.99) and 0.88 (0.79–0.94), respectively (Table 2). These indicate excellent and high test-retest reliability, respectively.

**Table 2:** Test-retest reliability of the Lower Limb Functional Index-10 Sections 1 and 3

	Time point 1	Time point 2	ICC (95 % CI)
	(*n* = 29, Section 1; *n* = 28, Section 3)	(*n* = 29, Section 1; *n* = 28, Section 3)	
Section 1, total score	3.53 (3.25)	3.55 (3.37)	0.98 (0.96 to 0.99)
Section 3, percentage	70.71 (32.85)	72.68 (31.22)	0.88 (0.79 to 0.94)

Section scores are presented as means (standard deviations) for patients who completed both sections at time points 1 and 2

*ICC* intra-class correlation coefficient, *CI* confidence interval

The MDC for Section 1 and 3 of the LLFI-10 was individually calculated using data collected from patients who completed the LLFI-10 at time points 1 and 2. Section 1 demonstrated a MDC of 1.27 points, whilst Section 3 demonstrated a MDC of 30.22 %.

### Internal consistency

**Fig. 2:**
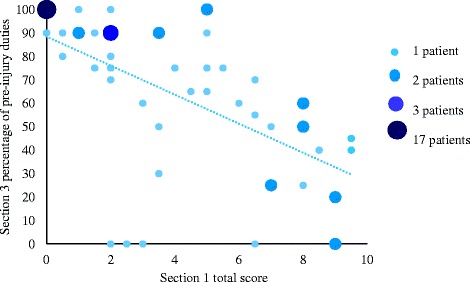
Correlation between Section 1 and 3 of the Lower Limb Functional Index-10 questionnaire

The correlation between Section 1 and 3 of the LLFI-10 was analyzed using data from the whole sample of patients at both initial and follow-up time points (*n* = 67). There was a high negative correlation between Section 1 total scores and Section 3 percentages of pre-injury duties, *r*_*s*_ = −0.83, *p* ≤ 0.001 (Fig. 2).

## Discussion

This study demonstrates that Section 1 and 3 of the LLFI-10 have excellent and high test-retest reliability, respectively, confirming that these two sections are reliable measures of lower limb function in patients with lower limb burns. This result also reflects that using a multi-item scale is preferred over using a single scale. As burns are both traumatic and complex injuries, an outcome measure that accounts for variations in pain is useful in evaluating outcome [16]. The results of the current study concur with a previous study involving the LEFS which demonstrated that the tool has excellent test-retest reliability with an ICC of 0.94 (95 % CI 0.89) in patients with lower extremity musculoskeletal conditions including hip osteoarthritis, knee meniscal injuries, and ankle/foot ligament sprains [23]. Therefore, the test-retest reliability of Section 1 of the LLFI-10 is similar to a previously established lower limb-specific questionnaire utilized in non-burn patient population groups. Furthermore, the test-retest reliability of Section 1 is also comparable to the English (ICC 0.92), Spanish (ICC 0.96), and Turkish (ICC 0.97) published versions of the LLFI [12–14].

Section 1 was shown to be responsive to a change of ≥1.27 total score points. As Section 1 is scored in 0.5-point increments, clinicians can be 95 % confident that a change of ≥1.5 points in Section 1 total score between measures indicates a real change in a patient’s lower limb function. It has previously been reported that Section 1 is responsive to a change of ≥1.67 total score points in patients with various lower limb musculoskeletal disorders, where clinicians can be 90 % confident that a change of ≥1.5 (7.87 %) total score points reflects a change in a patient’s lower limb function [24]. Thus, the current study suggests that the sensitivity of Section 1 in patients with burns is superior to what has previously been reported for other patient population groups. This is postulated to be due to a possibly broader variation in presentation and causes of injury, within a musculoskeletal population. Similarly, it has been shown that clinicians can be 90 % confident that the LEFS is responsive to a change of ±9 scale points out of a maximum of 80 (8.89 %) in patients with a range of lower extremity musculoskeletal conditions, excluding burn injuries [23]. Thus, the sensitivity of Section 1 of the LLFI-10 is comparable to that of the LEFS.

In comparison, clinicians can be 95 % confident that a change of ≥30 % on Section 3 reflects a real change in a patient’s function. These results suggest that the sensitivity of Section 3 of the LLFI-10 is relatively variable, allowing for greater effect of measurement error. To date, no previous studies have reported the sensitivity of Section 3 of the LLFI-10.

The current study demonstrates a high negative correlation between Sections 1 and 3 of the LLFI-10 reflecting the opposite scoring systems and also suggesting that there is a good level of association between the two sections. The results of this study suggest that although Section 1 and 3 scores may agree to a large extent, each section provides unique information and thus both may be useful within clinical settings. In addition, Section 3 requires patients to respond to one question only and is not a significant burden on patients. As previously discussed, however, Section 1 of the LLFI-10 is sensitive to changes in lower limb function in patients with lower limb burns whereas Section 3 demonstrates greater relative variability which supports the primary use of Section 1 within clinical settings in the future.

Overall, Section 1 and 3 of the LLFI-10 enable burn clinicians to accurately assess a patient’s lower limb function and utilize this information to identify and monitor specific aspects of a patient’s current alteration in function directing the patient’s treatment goals to maximize patient outcomes. Our group will now move forward and use these results to reduce patient measurement burden.

A strength of this study is that the majority of patients recruited had sustained minor burns so this is likely to improve the generalizability of the results obtained to the Australian burn patient population. A multi-center study may be warranted as the current study included only patients with lower limb burns in WA. The requirement for patients to return the follow-up questionnaire via mail due to WA being the largest state in Australia and having a vast catchment size area may have reduced the proportion of patients to complete the follow-up questionnaire. Face-to-face contact with patients at follow-up if and where possible may reduce loss of patients to follow-up. The limitations of the current study include the demonstration of a possible ceiling effect for 17 patients who completed the questionnaires at both time points. Additionally, patients with superficial dermal burns were included in the study which may lessen the impact of the results as superficial dermal burns have been shown to rapidly heal and result in minimal long-term sequelae [25].

In future studies, it would be useful to determine the minimal clinically important difference of Section 3 as an additional measure of the sensitivity of this tool to identify clinically relevant changes in the functional ability of a patient who has sustained a lower limb burn [26]. Furthermore, the clinical performance of the LLFI-10 including its validity, reliability, and sensitivity could be examined in patient populations with lower limb burns in other countries, particularly to ascertain whether the performance of the LLFI-10 differs if the majority of patients recruited have sustained major burns.

## Conclusions

This study demonstrates that Section 1 and 3 of the LLFI-10 questionnaire are reliable measures of lower limb function in adult patients with lower limb burns, and each provides information unique to the other. Furthermore, Section 1 is sensitive to changes in patient function and is thus likely to be of greater utility in clinical settings to measure the effects of interventions provided to patients with lower limb burns over time.

## Abbreviations

ADL: activities of daily living
ICC: intra-class correlation coefficient
ICF: International Classification of Functioning, Disability and Health
LEFS: Lower Extremity Functional Scale
LLFI: Lower Limb Functional Index
LLFI-10: Lower Limb Functional Index-10
MDC: minimal detectable change
QuickDASH: Shortened disability of the arm, shoulder and hand questionnaire
WA: Western Australia
